# Intraoperative Discovery of Lymphoma in the Knee During a Total Knee Arthroplasty: A Case Report

**DOI:** 10.7759/cureus.67943

**Published:** 2024-08-27

**Authors:** Myung-Jin Cha, Sanjay Kubsad, Mark Haft, Adam S Levin, Lucas E Nikkel

**Affiliations:** 1 Department of Orthopedic Surgery, Johns Hopkins University, Baltimore, USA; 2 Department of Orthopedic Oncology, Johns Hopkins University, Baltimore, USA

**Keywords:** dlbcl, tka, total knee arthroplasty, primary lymphoma of bone, diffuse large b-cell lymphoma

## Abstract

A 67-year-old male with a past medical history of coronary artery disease, hypertension, and obesity presented with severe left knee pain and severe tricompartmental osteoarthritis. After failing conservative treatments and completing a preoperative medical workup, the patient was scheduled for total knee arthroplasty. Intraoperatively, a pathologic fracture of the distal femur was discovered, and the procedure was aborted. Histopathologic evaluation of the femur fracture revealed diffuse large B-cell lymphoma. Intraoperative discovery of a pathologic fracture should be treated as an underlying malignancy until proven otherwise. In these cases, surgery should be aborted until definitive diagnosis and management can be planned.

## Introduction

Diffuse large B-cell lymphoma (DLBCL) is one of the most common non-Hodgkin’s lymphomas (NHLs) in the adult population in the United States [1]. However, primary lymphoma of the bone (PLB) is relatively rare, representing less than 1% of adult lymphomas, with only 37.5% of those cases occurring in the appendicular skeleton. When PLB does occur, the most common etiology is DLBCL [1,2]. DLBCL typically occurs in male patients over the age of 70 who present with a growing tumor mass involving lymph nodes, with up to 40% of patients experiencing extra-nodal involvement [3,4]. Commonly referred to as the B-symptoms, about a third of patients with DLBCL experience fever, weight loss, and night sweats, along with symptoms related to organ involvement depending on the location of the malignancy [5]. 

Before surgery, it is common for potential total knee arthroplasty (TKA) patients to obtain imaging of the operative knee and a thorough medical clearance, including routine blood work (complete blood count, comprehensive metabolic profile, hemoglobin A1c) and additional testing based on the patient’s individual comorbidities [6]. The goal of this preoperative clearance is to identify issues that may interfere with the operative plan and to medically optimize the patient to reduce complication rates [6]. Despite thorough medical clearance, unique medical problems can go unseen. Here, we present a case of aborted TKA due to a pathologic fracture of the distal femur found intraoperatively that was discovered to be DLBCL after histopathologic evaluation.

Statement of informed consent

The patient in this case report was informed and agreed that data concerning the case would be submitted for publication.

## Case presentation

This 67-year-old male presented in the outpatient setting with increasingly severe left knee pain ongoing for 18 months, without inciting injury, that had begun to impair his activities of daily living. The patient reported pain with activity and weight-bearing that required intermittent use of a cane to ambulate. He noted sharp, “burning” pain diffusely about the left knee that would occasionally wake him up at night. He had attempted but failed to achieve meaningful relief from acetaminophen, nonsteroidal anti-inflammatory drugs, diclofenac gel, and physical therapy. He had also received one intraarticular corticosteroid injection, which did not provide any pain relief. 

**Figure 1:**
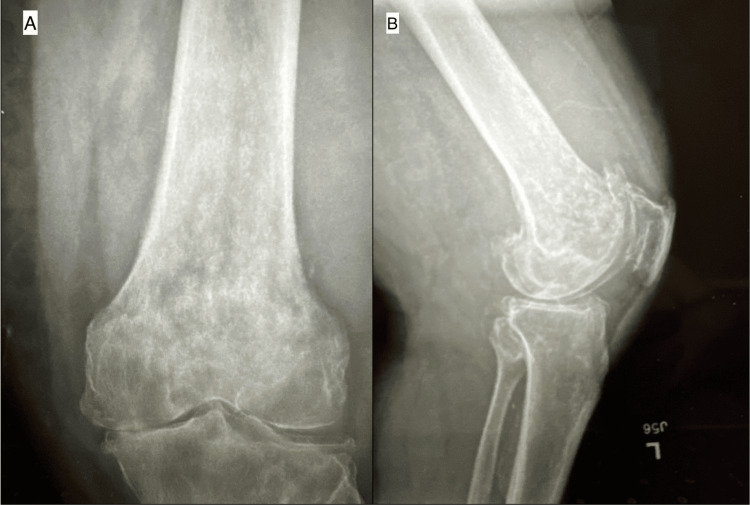
Preoperative (A) anteroposterior and (B) lateral knee radiographs.

His past medical history was significant for heart disease and a body mass index (BMI) of 31.2. He quit smoking in 2003 and had a 30-pack-year history. There was no previous surgical history of the left knee. Initial examination of the knee was notable for varus alignment, mild effusion, restricted range of motion (10°-85°), and tenderness to palpation in the medial and lateral joint lines. There was no varus or valgus laxity; however, the Lachman test was positive, with a 10 mm anterior translation of the tibia. Preoperative radiographs of the left knee were read by both the attending orthopedic surgeon and radiologist as severe tricompartmental osteoarthritis, most notably in the medial compartment, and a moderate joint effusion (Figure 1). 

On routine preoperative testing, he was noted to have hypercalcemia (calcium 11.3 mg/dL) and anemia (hemoglobin 12.6 g/dL), neither of which was present in any prior labs dating back four years. Because of these lab abnormalities, he was referred to hematologic oncology for further evaluation. After obtaining normal iron studies, the referring oncologist administered a single dose of denosumab for hypercalcemia and cleared the patient to proceed with TKA.

**Figure 2:**
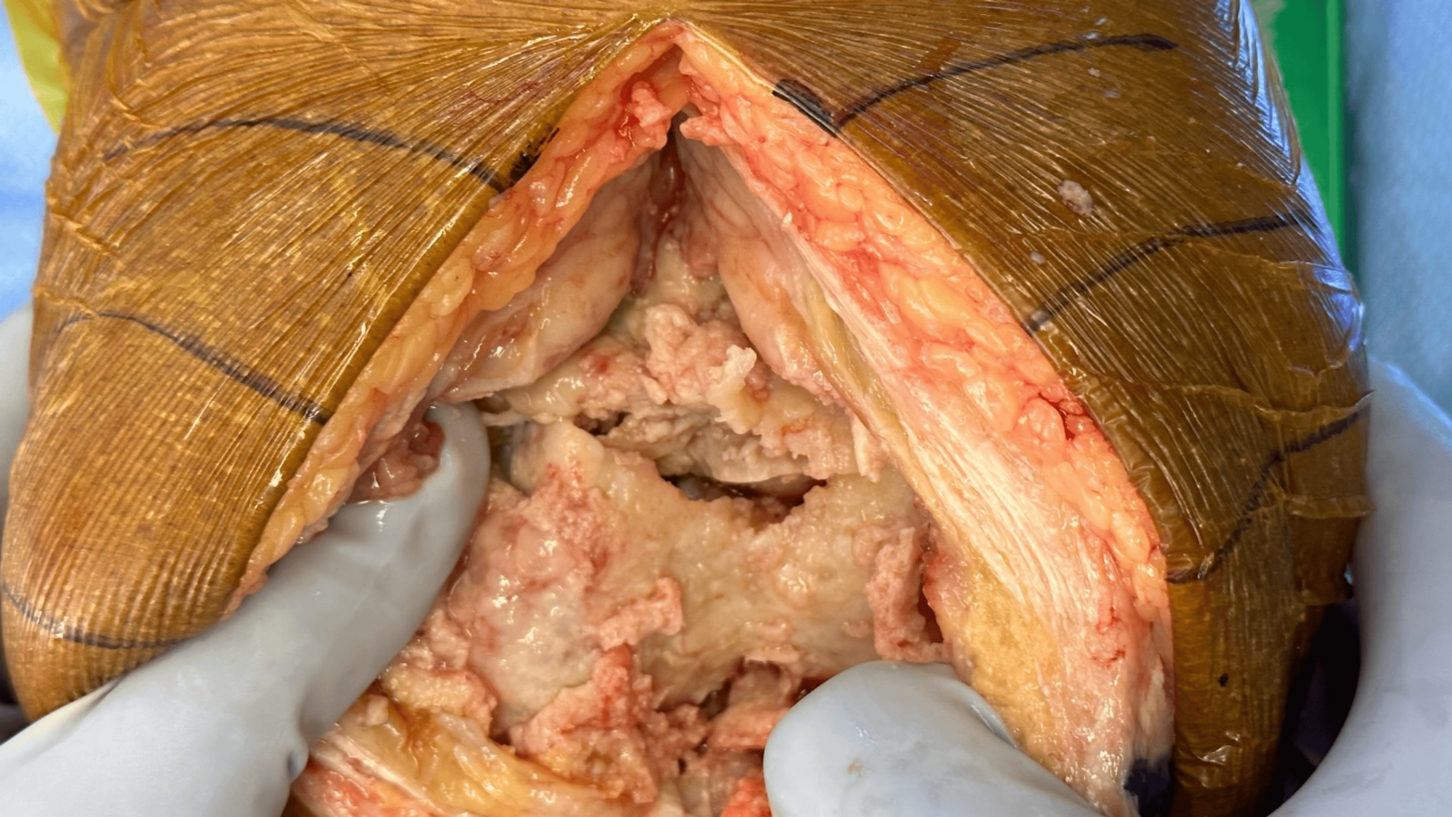
Photograph of distal femur fracture observed intraoperatively.

In the preoperative bay on the day of surgery, the patient noted increased knee pain and swelling requiring the use of a walker over the three weeks prior to his surgical date. Of note, the patient had a two-month gap between his initial consultation/X-rays and his surgical date due to a urinary tract infection, which was treated by his primary care physician. During this time frame, he had repeat lab work performed, which demonstrated the resolution of his hypercalcemia but continued anemia (hemoglobin 11.7 g/dL three days before surgery). No ESR and CRP were obtained preoperatively. The patient underwent routine preoperative evaluation from the nursing, anesthesia, and surgical teams. Intraoperatively, a nondisplaced left distal femur fracture was observed after the initial arthrotomy, and the TKA was aborted for further evaluation (Figure 2).

**Figure 3:**
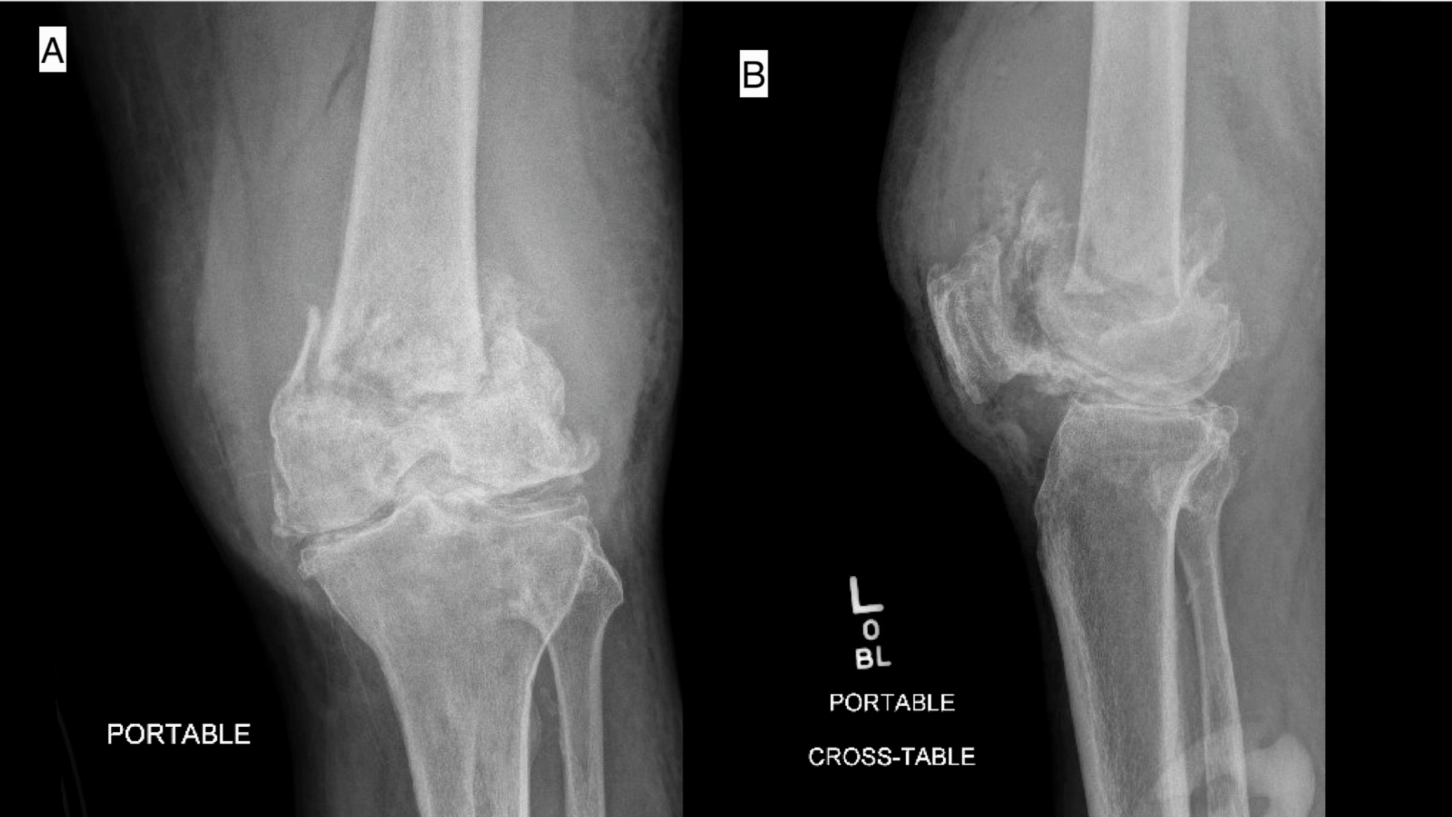
Knee (A) anteroposterior and (B) lateral radiographs taken immediately after aborted total knee arthroplasty.

After the aborted procedure, an initial workup, ordered by the orthopedic specialist, and consisting of CT imaging of the chest, abdomen, and pelvis demonstrated left internal iliac and inguinal lymphadenopathy. Radiographs and MRI of the left knee taken after the aborted surgery revealed a nondisplaced pathologic fracture of the distal left femur suspicious for neoplastic/metastatic disease (Figure 3).

The patient was reevaluated by hematologic oncology in the hospital, where it was revealed that in the month leading up to surgery, he had had 15 pounds of unintentional weight loss. The patient had initially believed this was due to healthier eating and not having an appetite because of the constant pain in his knee. An open femoral biopsy taken five days after the initial surgery revealed osteonecrosis without a viable tumor. 

**Figure 4:**
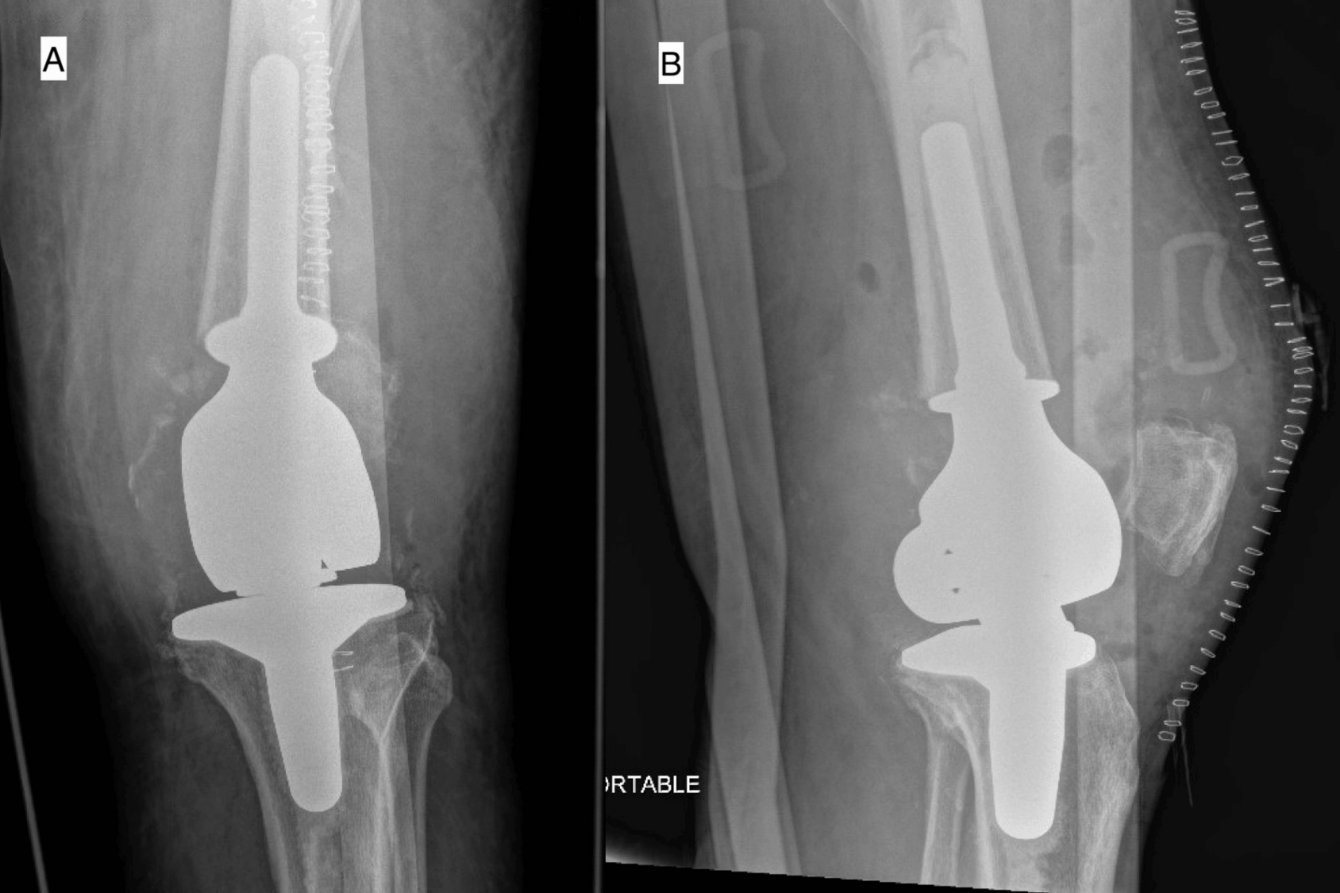
Distal femur replacement postoperative (A) anteroposterior and (B) lateral radiographs.

Three weeks after the initial, aborted surgery, the patient underwent an otherwise uncomplicated distal femoral resection and reconstruction with a distal femur-replacing total knee arthroplasty (Figure 4).

**Figure 5:**
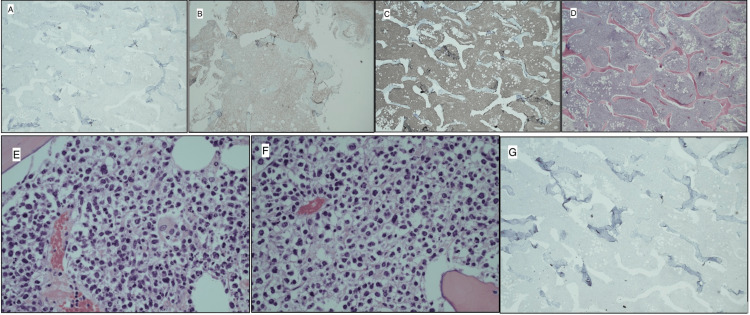
Histopathology slides of the pathologic bone. (A) CD3 immunohistochemical analysis in two times original magnification. (B) CD10 immunohistochemical analysis in four times original magnification. (C) CD20 immunohistochemical analysis in two times original magnification. (D) H&E stain in two times original magnification. (E) H&E stain in 40 times original magnification. (F) Another H&E stain in 40 times original magnification. (G) PAX5 immunohistochemical analysis in two times original magnification.

Pathology samples sent from this case were consistent with DLBCL. Histopathology slides showed diffuse lymphoid proliferation of bone. Large, atypical lymphocytes with irregular nuclear contours, vesicular chromatin, mitotic activity, and prominent nuclei were observed along with apoptotic bodies (Figures 5A-5G).

Per immunohistochemical analysis, the specimen was positive for CD20, CD79a, and PAX-5 (variable), with coexpression of CD10 and CD3 (Figures 5A-5G). The patient was treated with six cycles of polatuzumab, vedotin, and rituximab-cyclophosphamide, doxorubicin, and prednisone (CHP) chemotherapy, as well as local radiation therapy to the left knee. No lymph node biopsy was obtained. The most recent positron emission tomography/computed tomography (PET/CT) scan six months postop demonstrated substantially decreased size and metabolic activity of previously identified left para-aortic and pelvic wall soft tissue masses, left external iliac nodes, and left anterior medial upper thigh soft tissue mass. At his most recent follow-up, the patient reported doing well overall. He was in minimal pain, ambulated with a cane, and was working with physical therapy, and his knee range of motion was from 0° to 100°. 

## Discussion

This case is a rare presentation of an aborted TKA secondary to an intraoperatively recognized nondisplaced pathologic distal femur fracture. Although the patient’s chief complaint and history of present illness were mostly consistent with chronic osteoarthritis, in retrospect, there were multiple clues to the underlying pathology present during preoperative workup. The patient had symptoms consistent with B-symptoms, including night pain, weight loss, and swelling of the affected knee. In addition, it is likely that the patient’s preoperative anemia and hypercalcemia were caused by the underlying DLBCL. Previous research has demonstrated that, at diagnosis, DLBCL is associated with anemia in 39% of patients and hypercalcemia in 23% of patients [7,8]. Furthermore, patients with hypercalcemia of malignancy develop a higher degree of hypercalcemia over a shorter period of time than those who have hypercalcemia of other etiologies [9]. Thus, patients are further prone to expressing symptoms faster and should raise suspicion within the care team for malignancy [9]. 

To our knowledge, there are few reports of the diagnosis of DLBCL via the intraoperative discovery of a pathologic fracture during TKA. In 2008, Watson and Cross [10] described a case in which a bone cut from the distal femur during TKA was sent for histopathology after the surgeons had concerns about the appearance of the bone. The TKA was completed and subsequent pathology results came back as malignant lymphoma of B-cell origin. Other studies have detailed the findings of DLBCL in the synovium during TKA in which histopathologic testing was spurred by abnormal synovial appearance during surgery [3]. In both of these reports, preoperative workup was normal, and the diagnosis was made only by the decision to proceed with further testing based on abnormal tissue appearance. Additionally, Visser et al. [11] reported an incidental finding of NHL in the synovium of the knee of a patient who was to undergo TKA. As in the present case and that of Visser et al. [11], a fracture of the distal femur was not recognized preoperatively by the surgical team or radiology and was subsequently recognized intraoperatively. 

In addition to the abnormal appearance of tissue or bone, symptoms of NHL as a primary or metastatic manifestation in the distal femur can also mimic an infected knee with symptoms such as swelling and fever, which can further complicate the diagnostic and treatment plans for the care team [12]. 

## Conclusions

Our case highlights (i) the importance of maintaining a high index of suspicion for DLBCL in patients with chronic osteoarthritis with intraoperative discovery of fracture and (ii) some clinical cues that may suggest an underlying pathology other than osteoarthritis. When a patient presents with high-risk features such as blood and electrolyte abnormalities or a history consistent with B-symptoms (in our case, night pain, weight loss, and swelling of the affected knee), the surgical plan needs to be reassessed to investigate any other underlying etiology of knee pain. In cases of persistent arthritis that is non-responsive to anti-inflammatory drugs, thorough imaging and biopsy may be performed and a histopathologic analysis of tissue obtained intraoperatively may be warranted. Exploratory workup involving a multidisciplinary care team to rule out malignancy should also be conducted and a comprehensive postoperative care plan should be in place before the surgical intervention as well.
